# Tanner’s tempo of growth in adolescence: recent SITAR insights with the Harpenden Growth Study and ALSPAC

**DOI:** 10.1080/03014460.2020.1717615

**Published:** 2020-05-20

**Authors:** T. J. Cole

**Affiliations:** Department of Population, Policy and Practice Research and Teaching, UCL Great Ormond Street Institute of Child Health, London, UK

**Keywords:** Height, height velocity, puberty, growth curve analysis, SITAR, ALSPAC

## Abstract

**Background:** James Tanner emphasised the “tempo” of growth, i.e. the adolescent spurt as summarised by its timing (age at peak velocity or APV) and intensity (peak velocity, PV).

**Aim:** The paper applies the SITAR growth curve model to pubertal growth data with the aim of clarifying the growth pattern across multiple measurements and the spectrum of APV and PV.

**Subjects and methods:** Data for 7–20 years on ten anthropometric measurements in 619 children from the Harpenden Growth Study, and on height in 10410 children from the ALSPAC study, were analysed using SITAR (SuperImposition by Translation And Rotation). SITAR models pubertal growth as a mean curve with APV and PV fitted as subject-specific random effects, and a random measurement intercept.

**Results:** Mean APV for Harpenden girls and boys averaged 12.0 and 13.9 years across the ten measurements. PV expressed as percent per year lay in the narrow range 4–8%. Splitting the ALSPAC subjects into 9 by 5 APV and PV groups and fitting separate SITAR models to each group confirmed SITAR’s good fit while highlighting the spectrum of growth patterns.

**Conclusion:** SITAR works well to summarise pubertal growth. The disappointment is that Tanner did not live to see it in action.

## Introduction

The human adolescent growth spurt is a remarkable phenomenon seen in few other primate species. Occurring towards the end of the growth period, after height velocity has been falling steadily since early infancy, the velocity suddenly increases to a peak of around 10 cm/year in boys and 8 cm/year in girls, a growth rate not seen since late infancy. Then, just as suddenly, the rate falls to zero and growth ceases (Tanner 1962).

The pubertal growth spurt is unusual not only for its intensity but also for its timing. Peak height velocity in individuals can occur as early as 10 years of age in girls or as late as 17 years in boys. On average boys peak two years later than girls, and this, coupled with their greater peak velocity (PV), explains the sex difference in adult height – the two sexes are very similar in height at 10 years (Tanner 1962). The combination of the sharp change in velocity and the peak’s variable timing leads to an apparently chaotic pattern of height growth across individuals during puberty.

James ‘Jim’ M Tanner (1920–2010) was an eminent British auxologist (a term that he invented), whose contribution to the study of human growth throughout the second half of the 20th century was remarkably wide-ranging. He was perhaps most famous for his work on growth at adolescence, which was the title of his first of many books on growth (Tanner 1962). In it he explored pubertal growth in depth, comparing the changes in the different organ systems in the two sexes, and discussing how to measure the progress of puberty in individuals. He emphasised the concept of the *tempo of growth*, a measure of passing time in individuals relating to their pubertal status as quantified by their developmental age.

Developmental age is a measure of maturation and can be based on the appearance of the individual child’s bones, teeth or secondary sexual characteristics, including menarche in girls. They are all markers that over time progress through a series of consistent and well-documented developmental stages. The Tanner stages of pubic hair, breast development and genitalia have proved popular for assessing developmental age, as they can be assessed on a single occasion and require no specialised equipment (unlike the radiographic images required for bones or teeth) (Marshall and Tanner 1969, 1970).

Alternatively, developmental age can be based on the timing of growth landmarks such as peak height velocity. This however cannot be measured on a single occasion; it requires longitudinal data, with individuals measured repeatedly over time. The data then need to be analysed to generate the individual velocity curves and extract the ages at PV. Thus, as Tanner (1962) pointed out, there are two requirements to study growth: a cohort study of individuals followed over time and a trained anthropometrist to make the measurements.

In 1948 Tanner’s developing interest in growth led him to set up the Harpenden Growth Study, recruiting as his experimental officer Reginald ‘Reg’ H Whitehouse, straight out of the Royal Army Medical Corps (Tanner 1988). Whitehouse quickly established himself as an anthropometrist of the highest calibre, making hundreds of thousands of growth measurements (by Tanner’s own estimate), using equipment he himself designed, until his retirement in 1976.

Whitehouse also proved himself indispensable in analysing the growth data, meticulously plotting the points and drawing smooth curves through them. This work led to the seminal two-part 1966 Tanner, Whitehouse and Takaishi paper on height and weight growth reference charts, which has set the example for many subsequent studies on growth references (Tanner et al. 1966).

Later Tanner and Whitehouse (with statisticians Marubini and Resele) analysed the adolescent growth spurt in 55 boys and 35 girls from the Harpenden Growth Study, fitting logistic curves to height, sitting height, leg length, shoulder width and hip width for each individual (Tanner et al. 1976). The curves fitted well overall, and the ages at PV were highly correlated across measurements, but the corresponding velocities less so.

Analytical methods for growth data have advanced since these papers were written. However the underlying aim remains the same: to summarise mean growth as a smooth curve and to express individual growth patterns relative to the mean. A recently described growth curve model, SuperImposition by Translation and Rotation (SITAR) (Cole et al. 2010) is well suited to growth in puberty. Based on a group of individuals it estimates a mean growth curve and defines the individual curves relative to it in terms of their size, timing and intensity of growth. In particular, timing corresponds directly with the individual’s age at PV (APV), while intensity equates to PV, which means that applying SITAR is a simple way to categorise individuals in terms of their “tempo of growth”.

Tanner’s simple but elegant vision of pubertal growth was that individual growth patterns could be summarised largely by the timing and intensity of PV. Conveniently, the SITAR model makes this same assumption, and at the same time it assumes – as Tanner did – that conditional on timing and intensity, the underlying velocity curve is essentially constant in shape.

The aim of this paper is to celebrate the legacy of Tanner – and Whitehouse – by using SITAR to explore the adolescent growth spurt from two distinct perspectives: how it varies across ten distinct linear measurements from the Harpenden Growth Study, and how in detail the shape of the height velocity curve depends on timing and intensity, using data from the much larger Avon Longitudinal Study of Parents and Children (ALSPAC). The first perspective builds on Tanner’s paradigm whereas the second tests it, by seeing just how invariant the height velocity curve is to differences in timing and intensity.

## Subjects and methods

### Harpenden Growth Study

The Harpenden Growth Study consisted of 701 white British children, 282 girls and 419 boys, born between 1929 and 1965 (median 1949) and recruited from the Highfield Children’s Home, Harpenden, UK between 1949 and 1969 at ages between 0.9 and 20 years. They were predominantly children of manual workers or the lower middle class (Tanner 1981).

**Figure 1.**
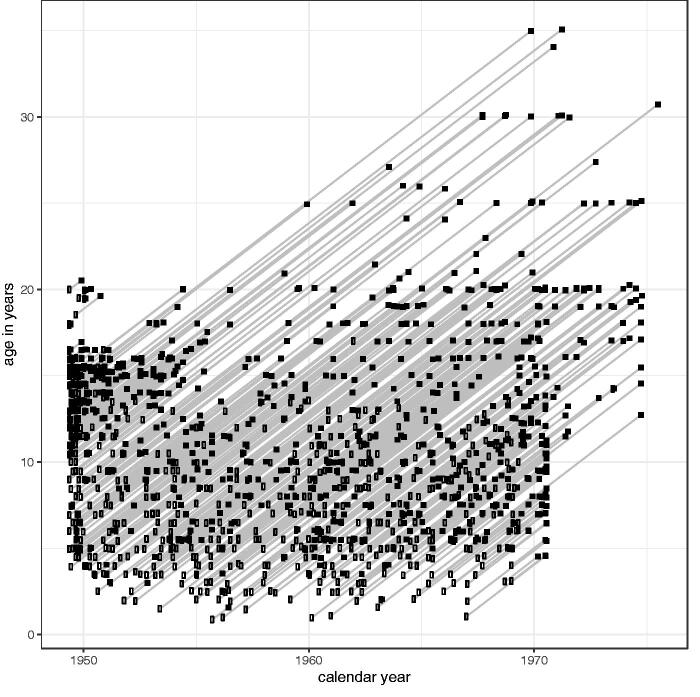
Lexis diagram for the Harpenden Growth Study, showing the ages and dates of measurement for 701 children. Each line represents one child, with their first (□) and last (▪) measurements marked.

They were measured 6-monthly outside puberty and 3-monthly during puberty, up to 35 years of age, on a total of 8097 distinct measurement occasions, 95% of them at ages between 4 and 19 years. Figure 1 is a Lexis diagram for the Study, with a slanting line for each child showing the date and their age at the time of their first and last measurements.

Whitehouse made all the measurements, including weight, height, other linear measurements and skinfold thicknesses. Here ten linear measurements are selected for analysis, ranked by median size: height*, sitting height*, leg length* (by difference), thigh circumference, shoulder width*, foot length, hip width*, arm circumference, knee width and elbow width (the asterisked measurements were also analysed by Tanner et al. (1976)). Note that the technical names for shoulder, hip, knee and elbow width used by Tanner were respectively bi-acromial, bi-iliac, bi-epifemur and bi-epihumerus width. All measurements were recorded to the last complete 0.1 cm. To focus on puberty the age range here is restricted to >7 to <20 years, during which period 619 children (248 girls and 371 boys) were measured on 6670 distinct occasions.

### Avon longitudinal study of parents and children (ALSPAC)

The Avon Longitudinal Study of Parents And Children (ALSPAC) consisted of over 15,000 children from Bristol UK recruited antenatally in 1990–1992 and followed up to age 20 (Boyd et al. 2013). Height was measured, to the nearest 0.1 cm, annually up to age 13, then at ages 15 and 17, by trained researchers. The present analysis involves 5183 girls and 5227 boys with 33122 and 31776 measurements aged from >7 to <20 years.

### Data cleaning

The two datasets had already been cleaned. For further cleaning SITAR models (see below) were fitted, and outliers were identified with standardised residuals exceeding 4 in absolute value (only 0.006% of data are this extreme based on a Normal distribution). There were very few outliers in the Harpenden Study, and they were inspected individually and either corrected (based on the context) or excluded. For the numerically larger ALSPAC models the outliers were excluded. Individuals with at least one measurement were included.

### Statistical methods

SITAR is a mixed effects growth curve model featuring a cubic spline mean curve and three subject-specific random effects that adjust the mean curve to best match the subjects’ own curves (Cole et al. 2010; Cole et al. 2014). The random effects are called size, timing and intensity, and each represents a simple transformation of the mean curve. *Size* indicates an up/down shift of the mean curve (in measurement units), reflecting how tall/short the individual is relative to the mean; *timing* reflects the individual APV (in age units e.g. years), seen as a right/left shift in the curve, positive for a later peak than average and negative for earlier; and *intensity* reflects the rate of passage through puberty as measured by PV (in fractional units), positive for faster than average and negative for slower. The degree of intensity shows itself by shrinking/stretching the age scale, which can be visualised as follows: imagine the image of a growth curve projected onto a half-open door. The effect of further opening the door is to make the curve appear narrower and steeper, corresponding to higher intensity, while closing the door makes the curve wider, shallower and lower intensity.

Algebraically the SITAR model is given by

(1) $$y_{ij}=\alpha_{i}+h\left[(t_{ij}-\beta_{i})e^{\gamma_{i}}\right]+\varepsilon_{ij}$$

where i=1…n subjects, j=1…N measurements, y and t are length (or other outcome) and age respectively, h[.] is a cubic spline function, α,β,γ are the normally distributed random effects size, timing and intensity, and ε is the residual assumed distributed as $N(0,\sigma_{r}{}^{2}).$

The smoothness of the natural cubic spline mean curve is controlled by the number of degrees of freedom (d.f.), each corresponding to a spline fixed effect, with the number chosen to minimise the Bayesian Information Criterion (BIC). In addition, there are fixed effects for size, timing and intensity, which ensure the corresponding random effects have mean zero. In nearly all the models here age was fitted after natural log transformation, to improve the fit, which effectively measures age differences in percentage units rather than years (Cole and Altman 2017). All the results were back-transformed to age for presentation purposes.

The output from each SITAR model consists of the mean curve as defined by the fixed effect coefficients, plus fixed effects for α,β,γ, subject random effects for α,β,γ summarised by their standard deviations and correlations, the residual standard deviation ε and the percentage of variance explained by the model, calculated as $100\left(1-(\sigma_{r}{}^{2}/\sigma_{f}{}^{2})\right)$ where $\sigma_{f}{}^{2}$ is the residual variance of the model fitted without random effects, i.e. with just the cubic spline mean curve. For models fitted with log age, the timing random effect SD was multiplied by median age to give units of age. The intensity random effect SD is age-invariant and essentially unaffected by age transformation.

The mean velocity curve was derived (in units of cm per year) as the first derivative of the mean curve plotted against age. In addition, four outputs were estimated from each mean velocity curve: the mean ages of PV and takeoff velocity (the minimum velocity prior to the peak), and the corresponding mean velocities. Note the mean curves are all plotted as length versus age, whether the SITAR model used age or log age. Standard errors for the outputs were obtained using the bootstrap.

For presentation purposes the curves were also compared in terms of percent velocity (i.e. in units of % per year), to adjust for the size differences between the measures. This was achieved by plotting length versus age with length on a 100 × natural log scale, and the first derivative of the curve is velocity measured in percent units (Cole and Altman 2017).

The aim of the ALSPAC analysis was to test the SITAR assumption that the underlying mean height curve is essentially the same shape for all individuals after adjusting for the timing and intensity of their growth spurt. To this end separate (global) SITAR models were first fitted for boys and girls, with log transformed age and 6 d.f. for the spline curves. Then individuals were split by sex into nine equal size groups based on the value of their timing random effects, i.e. APV, and separate “local” SITAR models were fitted to each group. As a second stage each APV group was split into five equal size groups based on individuals’ intensity random effects (PV) from the local APV model, giving 45 groups by sex, where in each group individuals were closely matched by timing and intensity. Again, separate local SITAR models were fitted to each group.

The group mean curves (either nine groups by APV or 45 groups by APV and PV) were then compared with the corresponding mean curves predicted from the global models, based on the mean timing and intensity random effects for each group. The numbers of nine APV and five PV groups were chosen to provide similar numbers per group of around 100, give more emphasis to timing than intensity, provide a median group by using odd numbers, and compromise between group number and group size. The models grouped by timing were fitted with 5 d.f. and the timing random effect was omitted as it was already adjusted for. The models grouped by timing and intensity also had 5 d.f., with both the timing and intensity random effects omitted as they were adjusted for.

All the analyses were carried out in *R* version 3.5.3 (R Core Team 2019), with SITAR fitted using the *sitar* package version 1.1.1 (Cole 2019).

## Results

### Harpenden Growth Study

**Figure 2.**
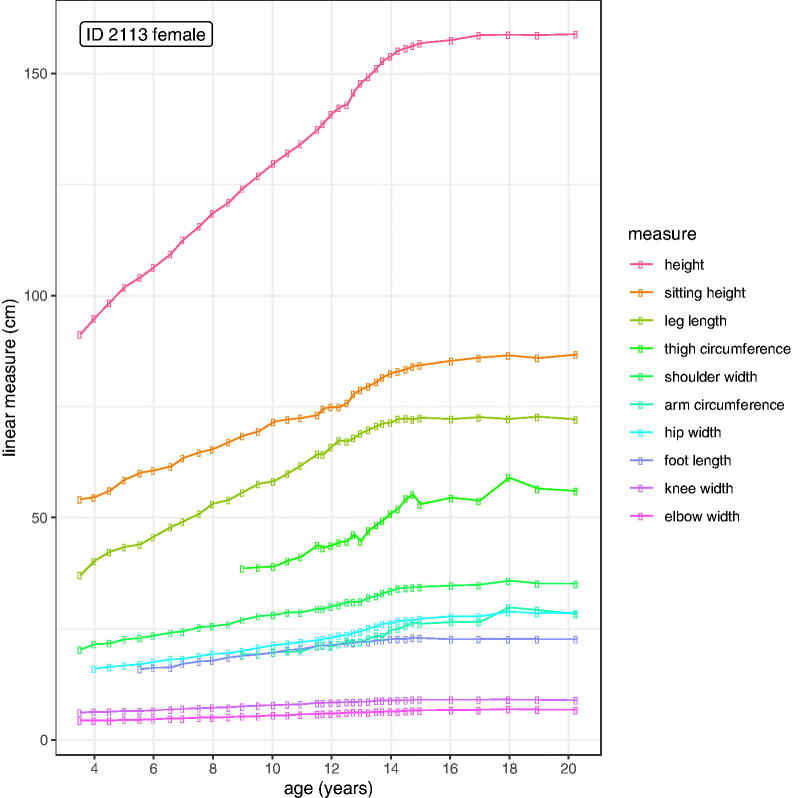
Data for one girl (ID 2113) from the Harpenden Growth Study between 3 and 20 years.

Figure 2 illustrates the anthropometry for one of the most intensely followed-up subjects, female 2113, born in 1947, with ten measurements on 36 occasions from age 3 to 20 years. The measurements vary in size from height (median 140 cm) to elbow width (median 5.9 cm), but all of them demonstrate a growth spurt at around 12 years. The regularity of the individual curves is striking, testimony to Whitehouse’s skill. Note that the curves for arm circumference and thigh circumference are noisier than the length curves, reflecting their soft mass component. Note also that leg length increases faster than sitting height prior to puberty but has a smaller pubertal growth spurt.

#### Mean growth curves

**Figure 3.**
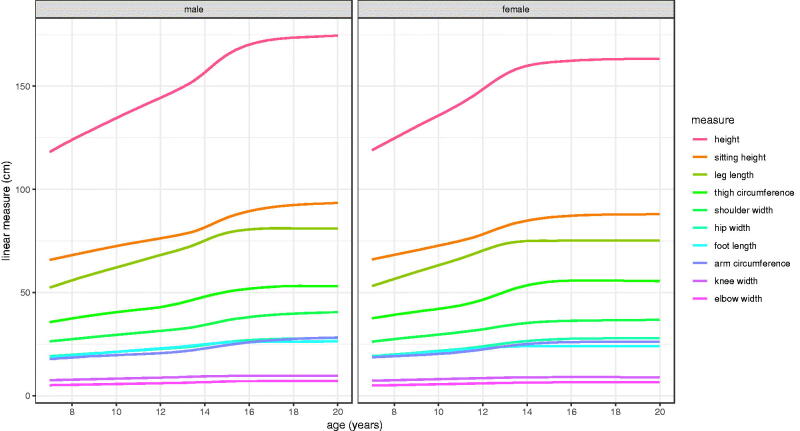
Mean curves for the Harpenden Growth Study SITAR length models by sex.

Separate SITAR models were fitted to the ten measurements by sex, a total of twenty models as summarised in Table 1. The number of measurement occasions per child ranged from 1 to 32 with median 8. The SITAR mean spline curves had between 3 and 7 d.f., with generally fewer for the smaller measurements. Figure 3 shows the fitted spline curves for the models grouped by sex, emphasising the broad similarity in shape, though the growth spurts for the smallest measurements are hard to see.

**Table 1.** Summary of the SITAR models fitted to ten linear measurements in boys and girls of the Harpenden Growth Study.

Measure	Sex	Subjects	Points	Median (cm)	df	Log age	Variance explained (%)	Residual SD (cm)	Residual CV (%)
Height	male	371	3847	147.6	7	yes	99.4	0.51	0.34
	female	248	2823	147.7	6	yes	99.5	0.45	0.31
Sitting height	male	371	3847	77.8	7	yes	98.2	0.45	0.58
	female	248	2822	78.3	5	yes	98.1	0.45	0.57
Leg length	male	371	3847	69.8	6	yes	98.8	0.45	0.64
	female	248	2822	69.1	6	yes	98.7	0.46	0.66
Thigh circumference	male	242	2552	43.8	5	yes	94.7	0.83	1.9
	female	180	1973	46.0	3	yes	93.1	0.99	2.1
Shoulder width	male	371	3846	32.1	7	yes	98.2	0.23	0.73
	female	248	2821	32.1	5	yes	98.0	0.23	0.72
Foot length	male	298	3076	23.3	7	yes	98.1	0.17	0.72
	female	191	2300	22.4	5	no	98.5	0.15	0.68
Hip width	male	317	3435	23.1	5	yes	98.7	0.17	0.74
	female	220	2593	23.6	6	no	97.7	0.22	0.92
Arm circumference	male	242	2552	21.3	4	yes	94.6	0.46	2.2
	female	181	1972	21.8	3	yes	91.6	0.56	2.6
Knee width	male	371	3846	8.9	6	yes	97.5	0.072	0.81
	female	248	2821	8.4	4	no	95.7	0.089	1.06
Elbow width	male	371	3847	6.2	6	yes	95.1	0.081	1.30
	female	248	2820	6.0	4	no	95.3	0.077	1.28

The models used a log age transformation for all except the four smallest female measurements, and the variance explained by the models ranged from 91% to 99.5%, again less for the smaller measurements and the circumferences. The residual standard deviations (SD) were largest for the large measurements, e.g. 0.5 cm for height, but divided by the median to give residual percent coefficients of variation (CV) they were the smallest (0.3% for height).

Table 2 summarises the random effect structure of the models. The SDs reflect the population variability in size, timing and intensity. The size SD is given in both absolute (cm) and proportional (%) units, using size at age 19 as denominator. On the percent SD scale the two sexes are very similar, with that for leg length (5.4%) unexpectedly larger than for sitting height (3.2%), while that for height (3.8%) is intermediate. The timing SD (variability in APV) is close to one year for all measurements. The intensity SD is generally around 0.1 to 0.2 (corresponding to 10% to 20% variation in PV), but somewhat larger for the smaller measurements and appreciably larger for the circumferences. The correlations in Table 2 are nearly all positive, with median values of around 0.4 for size-timing and timing-intensity, and 0.5 for intensity-size, indicating that larger individuals tend to have a later and more intense pubertal spurt. However the correlations are misleading as most are based on log age not age, which affects the correlations. For example, if boys height is refitted using age, the timing-intensity correlation of 0.28 changes to −0.28, indicating that timing and intensity are inversely correlated. Thus the true correlation between timing (as based on age) and intensity is negative not positive, and this applies quite generally in Table 2.

**Table 2.** Summary statistics for the SITAR model random effects in Table 1.

		Standard deviations				Correlations		
Measure	Sex	Size (cm)	Size (%)	Timing (yr)	Intensity (proportion)	Size-Timing	Timing-Intensity	Intensity-Size
Height	male	6.46	3.7	0.86	0.13	0.36	0.28	0.42
	female	6.21	3.8	0.87	0.12	0.24	0.32	0.38
Sitting height	male	2.95	3.2	1.06	0.17	0.39	0.36	0.33
	female	2.71	3.1	0.94	0.15	0.15	0.35	0.13
Leg length	male	4.47	5.5	0.85	0.13	0.44	0.27	0.54
	female	3.96	5.3	0.96	0.12	0.31	0.35	0.57
Thigh circumference	male	4.46	8.4	1.91	0.31	0.70	0.75	0.78
	female	3.77	6.8	1.28	0.35	0.43	0.57	0.73
Shoulder width	male	1.67	4.1	1.10	0.19	0.42	0.53	0.53
	female	1.50	4.1	1.03	0.15	0.19	0.44	0.40
Foot length	male	1.22	4.7	0.89	0.16	0.31	0.24	0.30
	female	1.19	4.9	0.92	0.17	0.01	−0.23	0.33
Hip width	male	1.55	5.6	1.04	0.19	0.46	0.42	0.69
	female	1.40	5.0	1.03	0.18	0.42	0.28	0.49
Arm circumference	male	1.97	7.1	1.53	0.36	0.40	0.36	0.67
	female	1.97	7.5	1.81	0.46	0.53	0.67	0.78
Knee width	male	0.44	4.5	1.00	0.27	0.37	0.40	0.50
	female	0.47	5.2	1.41	0.19	0.56	0.15	0.50
Elbow width	male	0.38	5.4	0.98	0.27	0.32	0.47	0.40
	female	0.34	5.3	1.21	0.22	0.11	−0.38	0.06

**Figure 4.**
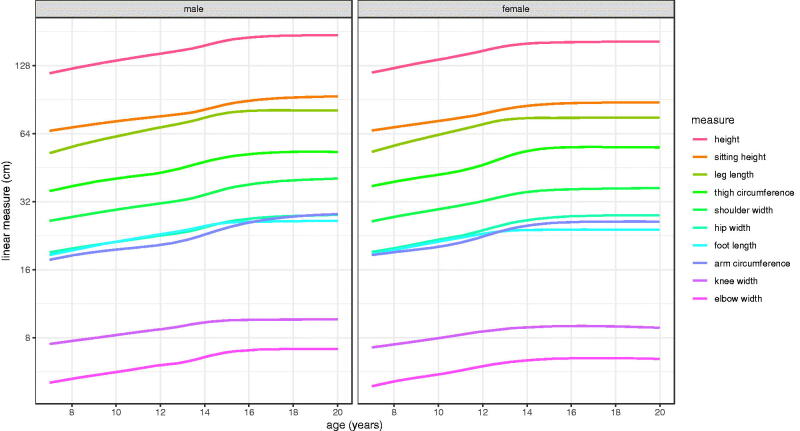
Mean curves for the Harpenden Growth Study SITAR length models by sex, with length on a log 2 scale to highlight the similarities in curve shape.

The model mean curves in Figure 3 are replotted in Figure 4 on a log 2 scale, i.e. constant rate of doubling, which scales the curves to be similar in shape, and in particular allows them to be compared across measurements by sex. For example, the height and shoulder width curves are seen to be very similar.

#### Mean velocity curves

**Figure 5.**
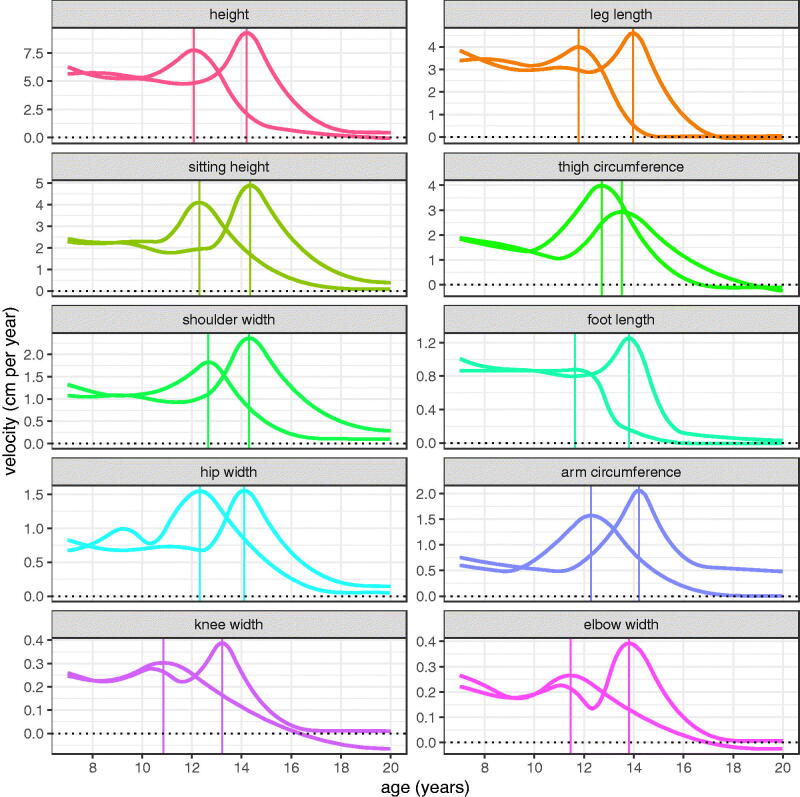
Mean velocity curves (cm/yr) for the Harpenden Growth Study SITAR length models by sex (girls left, boys right), with the ages at peak velocity indicated by vertical lines.

However, the best way to compare the growth spurts is via the corresponding velocity curves, as seen in Figure 5 by sex. Each measurement facet has its own scale, which enlarges the smaller measurement spurts. The corresponding APVs are highlighted as vertical lines, and the girls’ peaks are consistently about two years earlier than the boys’ (mean APVs across measurements 12.0 SE 0.18 and 13.9 SE 0.12 years for girls and boys). PV is generally slightly greater for boys than girls, with the striking exceptions of thigh circumference and to a lesser extent hip width.

**Figure 6.**
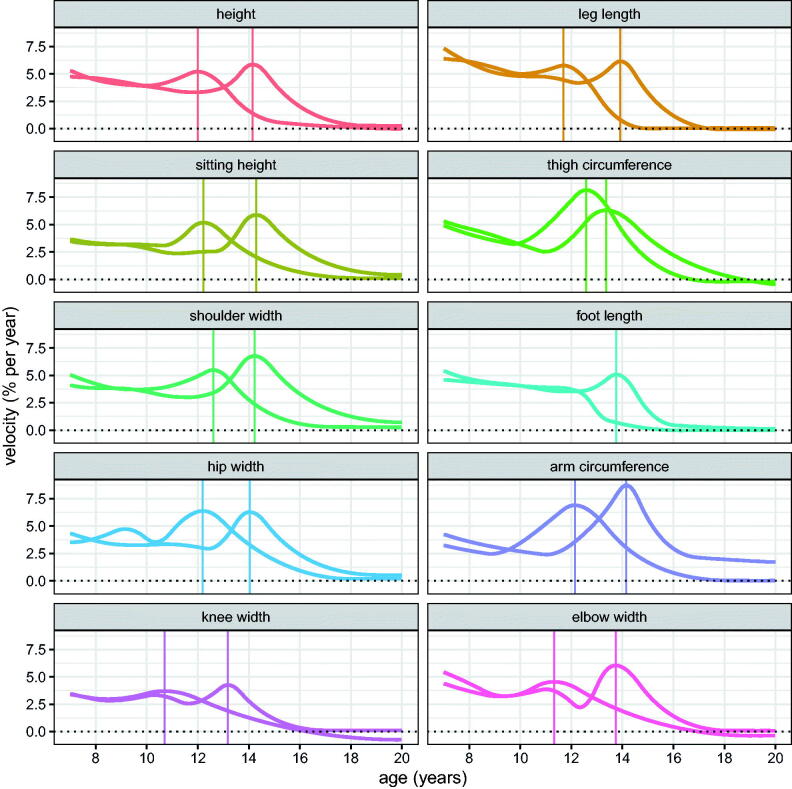
Mean percent velocity curves for the Harpenden Growth Study SITAR length models by sex (girls left, boys right), with the ages at peak velocity indicated by vertical lines.

Figure 5 is useful for comparing velocity curves in the two sexes, but less so for comparing across measurements, as the scales differ. The slopes of the curves in Figure 4 are directly comparable across measurements, being on a log scale, and as explained in the Methods, the slopes of these curves correspond to growth velocity measured in percent units. The corresponding percent velocity curves are shown in Figure 6, which make clear that peak percent velocity is not related to size; it is largest for boys arm circumference and girls thigh circumference, with both exceeding 8% per year, while the smallest velocity is for girls knee width (3.7% per year), a factor of just over two difference compared to the thirty-fold difference for linear velocity (boys height 9.3 cm/year versus girls elbow width 0.27 cm/year, Table 3). For the other measurements PV is consistently around 5% per year and slightly greater for boys than girls.

**Table 3.** Summary statistics for mean linear and percent velocity at takeoff and peak, and their mean ages, in the SITAR models.

Measure	Sex	Age at Takeoff (yr)	Age at Peak (yr)	Takeoff (cm/yr)	Peak (cm/yr)	Takeoff (%/yr)	Peak (%/yr)	Value at age 19 (cm)
Height	male	11.6	14.2	4.8	9.3	3.3	5.9	174.1
	female	9.9	12.1	5.3	7.7	3.9	5.2	163.3
Sitting height	male	11.1	14.4	1.8	4.9	2.4	5.9	93.0
	female	10.5	12.3	2.3	4.1	3.1	5.2	87.9
Leg length	male	12.2	14.0	2.9	4.6	4.2	6.2	81.1
	female	9.9	11.8	3.2	4.0	5.0	5.8	75.2
Thigh circumference	male	11.0	13.5	1.1	2.9	2.5	6.3	53.2
	female	9.7	12.7	1.3	4.0	3.2	8.2	55.6
Shoulder width	male	11.4	14.3	0.93	2.4	3.0	6.8	40.2
	female	9.1	12.7	1.1	1.8	3.7	5.5	36.7
Foot length	male	11.5	13.8	0.80	1.3	3.5	5.1	26.3
	female	10.3	11.6	0.86	0.88	*	*	24.0
Hip width	male	12.4	14.1	0.67	1.6	2.9	6.3	27.8
	female	10.3	12.3	0.78	1.5	3.5	6.4	27.9
Arm circumference	male	11.0	14.2	0.48	2.1	2.4	8.8	27.7
	female	8.8	12.3	0.48	1.6	2.5	6.9	26.1
Knee width	male	11.6	13.2	0.22	0.39	2.6	4.3	9.7
	female	8.3	10.8	0.23	0.30	3.0	3.7	8.9
Elbow width	male	12.3	13.8	0.13	0.39	2.2	6.0	7.1
	female	9.3	11.5	0.17	0.27	3.2	4.5	6.5

*no peak on % velocity curve.

As well as PV, a second landmark on the velocity curve is takeoff, the point where velocity is at a minimum immediately prior to the peak. Table 3 summarises the means for PV and takeoff velocity and their corresponding ages, both on the linear (cm per year) and proportional (% per year) scales as seen in Figures 5 and 6. The ages at takeoff/peak are very similar whether calculated on the cm or % scale, so those on the % scale are omitted. Bootstrap standard errors for APV range from 0.07 years for height and leg length up to 0.3+ years for the smaller measurements and the circumferences. For PV the standard errors are 0.12 cm per year for height and sitting height, 0.06 for leg length, and in between for the other measurements. Mean age at takeoff is two years earlier than mean age at peak, and earlier still for the circumferences. The takeoff velocities are about half the peak velocities on average, varying from a third or less for the circumferences to near equality for foot length in girls. The correlation across measurements between linear velocity at takeoff and at peak is high at 0.94, but for percent velocity the corresponding correlation is small and negative at −0.2. So the two are effectively unrelated when the scale differences are accounted for, showing that each measurement has its own distinct profile from takeoff to peak.

#### Random effects

An obvious question is whether individuals tend to be consistently large/small, early/late or fast/slow across all their measurements, and this can be tested for by looking at the correlations between the random effects. Across the ten measurements there are 45 correlations for each of size, timing and intensity in the two sexes, the median correlations being 0.49 (size), 0.46 (timing) and 0.37 (intensity) for boys and 0.45, 0.50 and 0.23 for girls. So within individuals, timing is conserved across measurements just as strongly as size is, while intensity is less so.

The order of measurements in each correlation matrix can be rearranged so the largest correlations migrate towards the diagonal – a process known as seriation – and this ranks the measurements in terms of their strength of association with their neighbours (see Supplementary Table 1). It effectively ranks them by their median correlation with the other measurements. Across the three random effects in the two sexes, height and leg length are consistently the most closely associated, followed by foot length and sitting height, while thigh and arm circumference are consistently the least associated, with the other four measurements in between. For example, the boys’ size random effects for height and leg length correlate at 0.87, whereas those for height and arm circumference correlate only at 0.16. Similar contrasts in correlation apply for boys’ timing and intensity random effects, and also for girls. So in terms of individual growth patterns, foot length is surprisingly similar to height and its two components, while the circumferences are very different.

### ALSPAC

**Figure 7.**
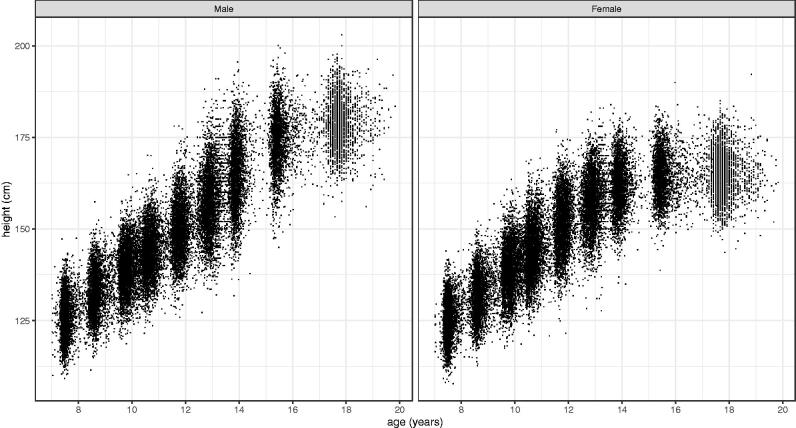
Height data for 5227 boys and 5183 girls in ALSPAC, collected over nine data sweeps between 9 and 17 years.

**Figure 8.**
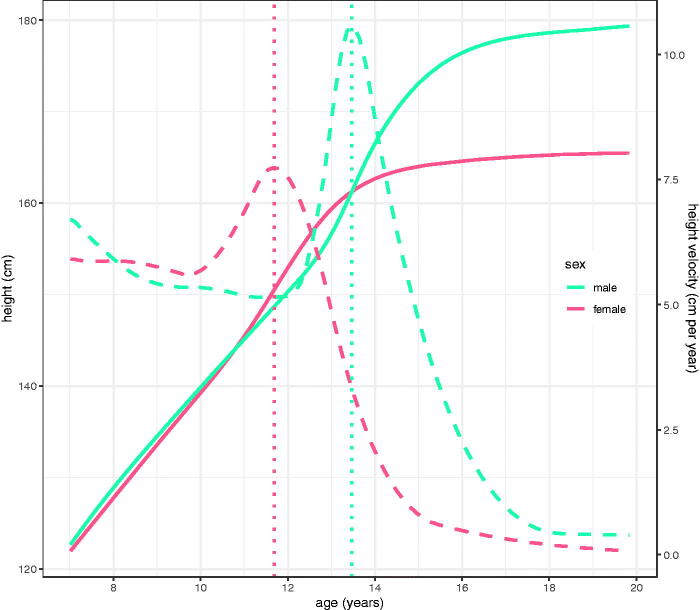
SITAR mean height and height velocity curves for ALSPAC by sex, with the ages at peak velocity indicated by vertical lines (girls left, boys right).

Figure 7 shows the height data for 5183 girls and 5227 boys in ALSPAC, where the nine data sweeps are clearly seen. The final sweep provides data from 17 to 20 years where the coverage is relatively sparse. Figure 8 illustrates the SITAR mean height curves and height velocity curves fitted to the data. On average girls are taller than boys between 10.8 and 13.5 years, while mean height velocity at age 19 is <0.1 and 0.4 cm per year in girls and boys, respectively. Table 4 summarises the two SITAR models, showing residual SDs twice those in Harpenden and correspondingly less variance explained. Mean APV is earlier than in Harpenden by 0.4 and 0.7 years (girls/boys), while PV is 0.0/1.3 cm per year greater and predicted height at age 19 is 2.1/4.9 cm greater. The bootstrap standard errors for mean APV and PV are around 0.02 years and 0.05 cm per year, respectively.

**Table 4.** Summary of the height SITAR models for boys and girls in ALSPAC.

Sex	Subjects	Points	Variance explained (%)	Residual SD (cm)	Residual CV (%)
male	5227	31776	98.0	1.02	0.34
female	5183	33122	97.8	0.90	0.31

	Standard deviations				Correlations		
Sex	Size (cm)	Size (%)	Timing (yr)	Intensity (proportion)	Size-Timing	Timing-Intensity	Intensity-Size
male	6.6	3.7	0.81	0.11	0.34	0.14	0.56
female	6.0	3.6	0.89	0.11	0.32	0.11	0.44

Sex	Age at Takeoff (yr)	Age at Peak (yr)	Takeoff (cm/yr)	Peak (cm/yr)	Takeoff (%/yr)	Peak (%/yr)	Height at age 19 (cm)
male	11.5	13.5	5.1	10.6	3.4	6.6	179.0
female	9.8	11.7	4.1	7.7	4.1	5.2	165.4

Tables 5 and 6 explore how age at peak height velocity affects the mean growth pattern. For this, individuals were split into nine equal size groups according to their timing random effect, and SITAR models were fitted to each group by sex. Thus the groups represent the spectrum from early to late puberty. Table 5 shows that the groups all have the same number of ∼580 individuals, but the early and late groups have consistently more data points and are also consistently more variable (with less variance explained, larger residual SDs, and larger size and intensity random effect SDs) than the central groups.

**Table 5.** Summary of height SITAR models for boys and girls in ALSPAC split into nine groups by age at peak height velocity.

Sex	Group	Subjects	Points	Variance explained (%)	Residual SD (cm)	Size SD (cm)	Intensity SD (proportion)	Size-intensity correlation
male	1	581	4305	95.5	1.4	6.5	0.12	0.51
	2	581	3926	97.6	1.0	6.5	0.12	0.43
	3	581	3383	97.1	1.0	6.0	0.10	0.49
	4	580	2889	96.9	1.0	5.0	0.08	0.51
	5	581	2506	96.2	1.1	4.4	0.08	0.45
	6	581	2867	96.6	1.1	5.2	0.07	0.49
	7	580	3277	97.1	1.1	6.4	0.08	0.55
	8	581	4137	97.1	1.1	6.3	0.09	0.55
	9	581	4486	96.4	1.2	5.8	0.10	0.59
female	1	576	4087	95.9	1.2	6.0	0.12	0.36
	2	576	3959	97.6	0.9	5.9	0.09	0.47
	3	576	3641	97.9	0.9	6.0	0.10	0.40
	4	576	3000	97.6	0.8	4.9	0.09	0.39
	5	575	3053	97.9	0.8	4.8	0.09	0.48
	6	576	3280	98.0	0.8	5.3	0.10	0.38
	7	576	3513	98.0	0.8	5.8	0.10	0.42
	8	576	4115	97.6	0.9	5.8	0.10	0.37
	9	576	4474	96.3	1.1	5.8	0.11	0.51

**Table 6.** Summary of the height SITAR models for boys and girls in ALSPAC split into nine groups by age at peak height velocity.

Sex	Group	Age at Takeoff (yr)	Age at Peak (yr)	Takeoff (cm/yr)	Peak (cm/yr)	Height at age 19 (cm)
male	1	9.2	12.5	5.8	9.5	176.7
	2	11.0	12.8	5.4	10.4	178.9
	3	10.9	13.0	4.8	9.8	179.7
	4	10.6	13.1	4.5	8.7	179.8
	5	10.4	13.1	4.4	8.1	179.5
	6	10.6	13.4	4.2	8.0	177.8
	7	10.7	13.7	4.1	8.0	177.7
	8	10.9	14.2	4.1	8.2	180.1
	9	11.0	15.1	4.2	7.4	181.7
female	1	8.5	10.1	6.4	8.1	163.0
	2	8.7	11.1	6.1	7.9	164.4
	3	8.9	11.2	5.9	8.0	165.0
	4	9.0	11.4	5.6	7.8	165.6
	5	9.6	11.5	5.5	7.5	165.8
	6	9.3	12.0	5.4	7.3	165.5
	7	9.1	12.4	5.3	7.6	165.2
	8	9.4	12.7	5.3	7.9	166.4
	9	11.1	13.2	4.6	7.4	167.0

Table 6 confirms that the groups are clearly ranked by mean APV, with 2.5 to 3 years between the earliest and latest groups. Age at takeoff shows a weaker trend, particularly in boys, but velocity at takeoff is strongly and inversely related to APV in both sexes. PV also falls with increasing APV. Those maturing late are consistently taller at age 19, by 4 cm in girls and 5 cm in boys compared to the earliest maturers.

**Figure 9.**
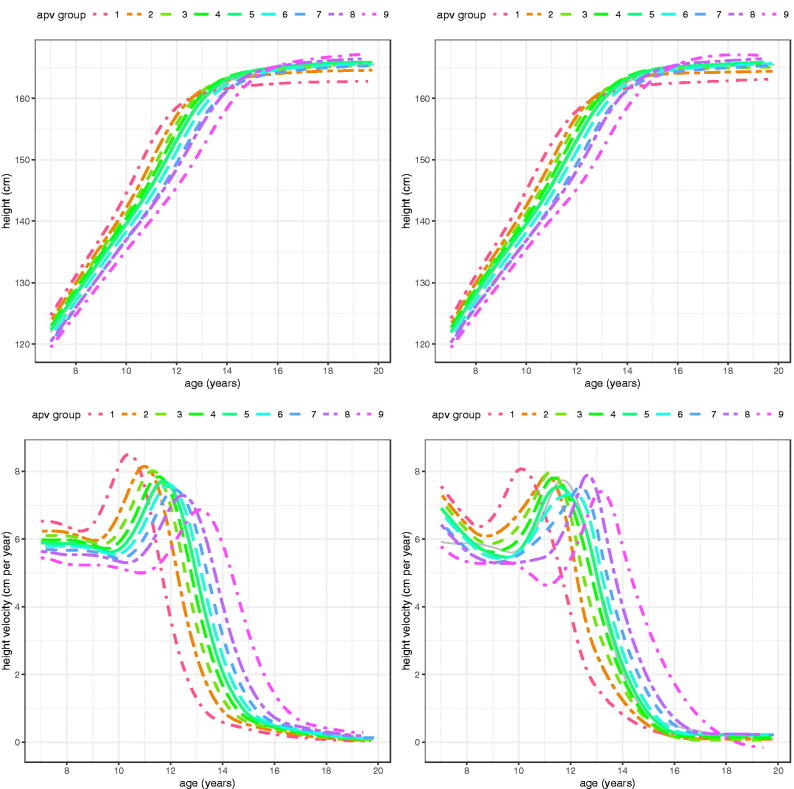
SITAR mean height and height velocity curves for ALSPAC girls, split into nine groups by age at peak height velocity. The left panels are predictions from the global model, while on the right are the group-specific mean curves.

Figures 9 (girls) and 10 (boys) visualise the mean height and height velocity curves for the groups in Tables 5 and 6. They are derived in two distinct ways – a) as predicted by the global SITAR models based on the group mean random effects for size, timing and intensity, and b) as fitted by the local group-specific SITAR models.

**Figure 10.**
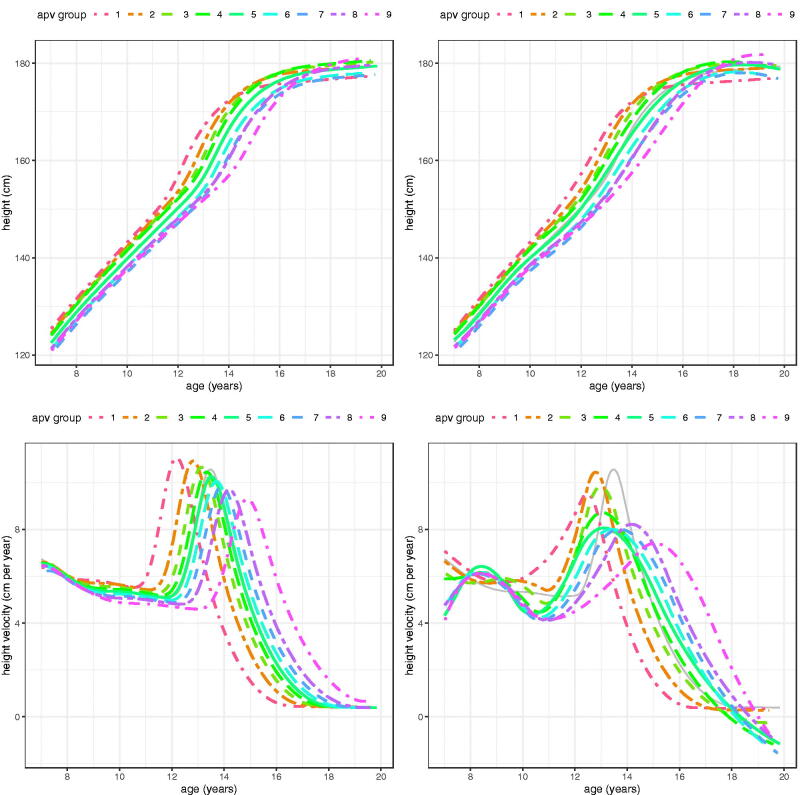
SITAR mean height and height velocity curves for ALSPAC boys, split into nine groups by age at peak height velocity. The left panels are predictions from the global model, while on the right are the group-specific mean curves.

The global and local height curves in Figures 9 and 10 are very similar in shape, indicating that SITAR has done a good job of predicting mean height by age across the spectrum of APV. However the height velocity curves are less so; the global curves are very consistent in shape and show a clear trend downwards with increasing APV (this trend arises from the use of a log age transformation, and had age been used instead predicted PV would be essentially the same across groups). The local curves, in contrast, vary considerably in terms of velocity and curve shape near the peak, but also in the shape of the curve prior to takeoff.

The global velocity curves show velocity falling slowly until takeoff, whereas with the local curves velocity prior to takeoff falls dramatically in girls and rises then falls in boys. This subsidiary peak in boys around age 9 years can also be seen in the height curves, where there is an upward “bulge” for the later groups. The reason why these patterns are not reflected in the global curves is because they feature in only some of the groups, and averaged across groups they disappear.

**Figure 11.**
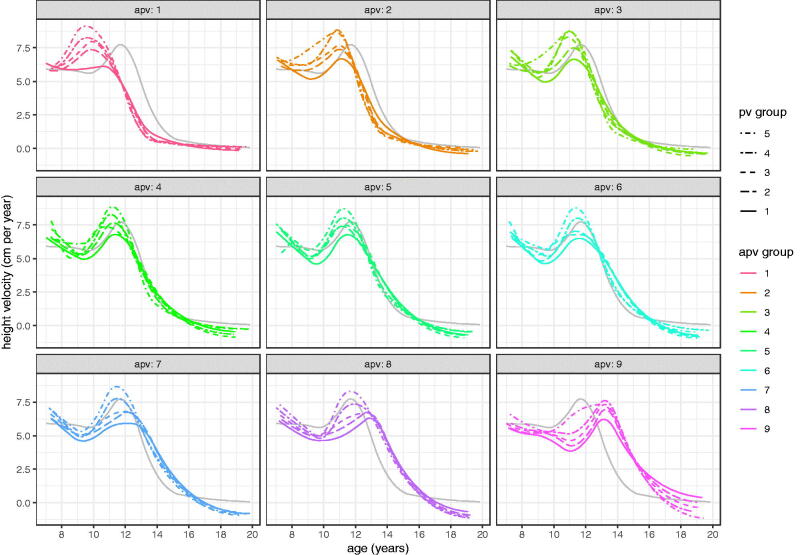
SITAR mean height velocity curves for ALSPAC girls, split into nine groups by age at peak height velocity (APV) and in turn five groups by peak height velocity (PV).

It is possible that the differences in velocity, particularly prior to takeoff, arise from sampling error and are not biologically meaningful. To test this the nine APV groups are each split into five further groups by individual intensity random effects i.e. PV, so splitting them into independent groups of faster and slower growers with the same APV. Figures 11 (girls) and 12 (boys) show the local velocity curves for these 9 × 5 = 45 groups by sex, where to aid navigation, each facet shows the global mean velocity curve in grey. The figures confirm two things; that mean PV is greater in those whose intensity random effect is greater, and that velocity prior to takeoff does indeed vary systematically in shape according to APV. This is particularly marked for boys, where the facets for the first two APV groups show no sign of a pre-takeoff peak, whereas the later facets show an increasingly obvious peak at around age 9 years, largely independent of APV. For girls there is just the hint of a pre-takeoff peak in the latest maturing facet. Each of the 45 models in these figures is based on ∼115 individuals, providing strong independent evidence for the existence of a “midgrowth” spurt at age 9 in later-maturing boys.

## Discussion

### Study findings

The study has confirmed the complexity of linear growth in puberty, driven as it is by individual variation in – Tanner’s term - the “tempo of growth”. This shows itself as variability in the intensity and timing of growth, i.e. PV and the age when PV occurs. The results show that the SITAR growth curve model works well with growth in puberty, because it explicitly estimates these two parameters in individuals, along with a third parameter representing their mean size. Simpkin et al. compared several alternative growth curve models for height in puberty, and they also concluded that SITAR was useful in providing unbiased estimates of age at peak height velocity (Simpkin et al. 2017).

But that said, the more detailed ALSPAC analysis has brought to light the existence of a midgrowth spurt at around 9 years in the later-maturing boys. By its nature it is hard to see unless the data are sufficiently numerous to disaggregate.

### Harpenden Growth Study

The Harpenden Growth Study is an important resource, not only for the biological insights arising from the data, but also for the way they demonstrate Reg Whitehouse’s auxological skill. The Lexis diagram (Figure 1) shows how Tanner and Whitehouse set about obtaining the Study children they needed, with many recruited at the outset in 1949 and followed up for 10 or more years, while other older early recruits dropped out soon afterwards. Tanner and Whitehouse continued to recruit children steadily over the next twenty years, typically at ages between 4 and 13 years, and Tanner went on to use the Study data in several seminal papers (Tanner et al. 1966; Marshall and Tanner 1969, 1970; Tanner et al. 1976).

#### Mean growth curves

The good fit of SITAR is demonstrated by the proportion of variance it explains. Taking as a baseline the data treated cross-sectionally, i.e. ignoring the repeated measures component, and then adding the three SITAR random effects, explains over 90% and up to 99.5% of the variance (Table 1), leaving a small component corresponding to the residual SD. In Harpenden the residual SD ranges from 0.5 cm for height down to 0.08 cm for knee and elbow width, while proportionally height is the least noisy of the measurements, its residual CV being only 0.3% versus 1.3% for knee and elbow. This reflects the fact that variability consists of two components: biological variation which scales with the measurement (i.e. the SD is proportional to size), and measurement error which is scale-invariant (in cm units) and hence relatively larger for small measurements. For height in ALSPAC the residual SD was 1.0 cm, twice that in Harpenden, reflecting the trade-off in measurement quality between a small study with a single (excellent) auxologist and the necessarily larger-scale and hence noisier measuring regime in a cohort the size of ALSPAC.

An obvious strength of the Harpenden Study is the opportunity it provides to compare the growth patterns of ten distinct linear measurements, ranging in size from height (median 148 cm) to elbow width (6 cm), a thirty-fold range (see Tables 1–3 and Figure 3). Tanner also analysed five of the measurements in 55 boys and 35 girls (Tanner et al. 1976), tabulating mean (SD) age at takeoff, APV, PV and final size by sex. Despite the smaller sample size his results were very similar to those in Table 3; final size agreed to within 0.3 cm except for boys’ height (0.5 cm), and similarly for APV to within 0.3 years. However, his correlations between the APVs of the five variables were implausibly larger – median 0.92 and maximum 0.97 - than the equivalent correlations for the timing random effects in Supplementary Table 1.

#### Mean velocity as a percentage

It has already been noted how variability scales across the measurements, but comparing the growth curves is complicated as the peak velocities are so different (Table 3). Plotting the data on a log scale (Figure 4), which converts velocity to percentage units, is a simple way to adjust for the differences in scale and allows the growth curves to be compared directly. The spacings between the measurement curves are similar in boys and girls, the one obvious exception being thigh circumference which is relatively larger for girls after puberty.

Figure 6 compares velocity in percentage units in the two sexes. Expressing velocity this way is novel, the more usual units being cm per year, but it is particularly useful here for comparing measurements on the different scales.

Figure 6 and Table 3 show that peak percent velocity is strongly conserved, with all except arm and thigh circumference in the narrow range of 4–6% per year, on average about 0.5% greater for boys than girls. However Figure 6 and Table 3 also show that the shapes of the velocity curves differ in detail between measurements, with sex differences in PV, APV and age at takeoff making each measurement profile unique.

Sitting height and leg length provide an interesting contrast in growth pattern. Prior to puberty, leg length grows fastest and sitting height slowest in both sexes. So during this period leg length increases progressively as a proportion of height. The two are similar in PV at around 4/5 cm per year in girls/boys, but at takeoff sitting height velocity is 1 cm per year lower than for leg length (Figure 5). Figures 3 and 5 show that sitting height continues growing well after leg length growth has ceased, and in boys it continues to age 19 and beyond. Also the size random effect SD is appreciably larger for leg length than sitting height (5.4% vs 3.2%, Table 2), so there is greater variability in leg length. Thus the two components of height grow in rather different ways during childhood.

The two circumferences, thigh and arm, behave differently from the eight lengths and widths. They have the largest percent peak velocities (for arm in boys and thigh in girls) and amongst the smallest velocities at takeoff. So the intensity of the circumference growth spurt (i.e. the increase in velocity from takeoff to peak) is particularly high, presumably due to the fact that it measures not just bone but also soft mass, which accumulates faster than bone. This fits with the residual CVs and the intensity random effect SDs for the circumferences being more than twice those for the other measurements (Tables 1 and 2).

#### Comparing random effects

Thus far the discussion has focussed on the shapes of the SITAR mean curves and velocity curves. The other component of the SITAR model is the set of subject random effects that indicate how the growth curves of individuals differ from the mean curve. Table 2 shows that the SD for the size random effect is more uniform when presented as the percent CV rather than the SD in cm units. This represents the variability of each measurement once timing and intensity have been adjusted for, so it reflects the variability when growth has ceased i.e. in adulthood.

This population CV is equivalent to that estimated for growth references using the LMS method (Cole and Green 1992), where the CV is the “S” in “LMS”. Thus for height in the British 1990 reference (Cole et al. 1998) the S value at age 19 is 3.9% for boys and 3.7% for girls, closely similar to the 3.7%/3.8% in Table 2. The corresponding ALSPAC values are 3.7% and 3.6% (Table 4). Dangour’s leg length and sitting height growth references (Dangour et al. 2002) give S values at age 19 of 5.4% for leg length and 4.6% for sitting height (averaged by sex), agreeing well with the CV for leg length but not for sitting height in Table 2 (5.4% and 3.2%). Tanner’s analysis of a subset of the Harpenden data gave CVs for adult size broadly similar to the size CVs in Table 2 (Tanner et al. 1976). In Harpenden sitting height is by far the least variable of all the measurements, whereas leg length is amongst the most variable (excepting the circumferences).

The timing random effect SDs in Table 2 are all (again with the exception of the circumferences) close to one year, the median being 1.0 years. For height they are 0.86/0.87 years in the two sexes, matching closely Tanner’s experience – “The SD of age at PHV is a little less than 1 year in nearly all published series; we have taken the value 0.9 years” (Tanner and Davies 1985). In ALSPAC the two SDs are slightly more varied at 0.81 and 0.89 years (Table 4).

The intensity random effect reflects the individual’s proportional difference in PV relative to the mean. So the intensity SD is effectively the CV for PV, or multiplied by 100 the percent CV for PV. The values for the intensity SD in Table 2 show it to be related to size, with the smallest values for height and leg length (0.12–0.13) and the largest (excepting the circumferences) for knee width and elbow width (0.2–0.3).

With multiple measurements available, it is obvious to ask if individuals tend to be similar in their random effects across measurements, i.e. do they tend to be consistently large/small, or early/late, or fast/slow in their growth pattern. This is addressed by looking at the correlations between measurements for each random effect by sex, as seen in Supplementary Table 1. The variables in the correlation matrices are sorted to put highly correlated measurements next to each other, which has the effect of migrating large coefficients towards the diagonal and small correlations away from it. The most strongly associated measurements are height and leg length (unsurprisingly, as leg length is a component of height) but more surprisingly the next most associated measurement is not sitting height but foot length (it ranks third three times and fourth twice in Supplementary Table 1). Why such a distal measurement should be closely correlated with height is something of a mystery – perhaps it represents a link between leg length and foot length. As expected from what has gone before, the two circumferences are consistently last in the rankings, being similar to each other but different from all the other variables.

### ALSPAC

The ALSPAC study design involved nine data sweeps spaced one to two years apart (Figure 7). This has been shown to be close to the optimal design for studying pubertal growth, as annual or biennial measurements minimise the data collection while retaining relevant information on the mean curve and subject random effects (Cole 2018). On these grounds one could argue that the 3-monthly measurement regime used in Harpenden was unnecessarily intensive.

The SITAR models for ALSPAC by sex appear in Figure 8 and Table 4. They can be compared directly to the SITAR models described by Frysz et al. (2018) which were fitted to a selected subset of ALSPAC data from age 5 to 20 years. Thus they had 45,065 measurements on 2688 boys and 3019 girls, as against 64,898 points, 5227 boys and 5183 girls here (Table 4). Despite the disparities in sample size their mean (SD) APVs were very similar to those here: girls 11.7 (0.8) and boys 13.6 (0.9) years, as against 11.7 (0.9) and 13.5 (0.8) years (Table 4). And similarly for PV: 7.7 (0.8)/10.0 (1.1) there and 7.7 (0.8)/10.6 (1.2) here (Table 4). The percentage of variance explained was close to 98% in all four models, and the two sets of random effect SDs were also very similar. Frysz et al. restricted their sample to individuals with at least one measurement in each of the age groups 5-<10, 10-<15 and 15 < 20 years. The fact that the results are effectively the same without applying this filter indicates that the filtering is unnecessary, and that results based on the whole sample are unbiased. In addition, the increased numbers allow for a mean curve with 6 d.f. rather than the 5 used by Frysz, which may explain the greater boys PV here of 10.6 cm versus 10.0 cm per year.

Comparing mean predicted adult height in ALSPAC and Harpenden demonstrates the secular trend in height from 1949 to 1991, a period of 42 years between their median dates of birth. Height at age 19 increased by 5 cm in boys and 2 cm in girls over that time, corresponding to trends of 1.1 and 0.5 cm per decade respectively. However the Harpenden children were of lower social class than in ALSPAC, and this may have exaggerated the trend. For comparison trends from the Flemish 2009 growth reference were 1.2 and 0.8 cm per decade (Roelants et al. 2009), and for the Finnish 2011 reference 0.6 cm per decade in both sexes (Saari et al. 2011).

A strength of SITAR is its ability to sort individuals by APV (Simpkin et al. 2017). Thus it is easy to use the global model to split the ten thousand ALSPAC children into nine groups by sex, based on their timing random effects, and then analyse these local groups separately. The results in Tables 5 and 6 and Figures 9 and 10 confirm that the local group curves (top right in each figure) are not only correctly ranked by mean APV, but are also very similar in shape to the curves predicted from the global models (top left).

Table 5 shows that despite having equal numbers of subjects, the central groups have fewer measurements and are generally less noisy than the more extreme groups. This is due to the group membership being based on individual timing random effects, and individuals with relatively few points have their random effect shrunk towards the mean of zero (in the extreme case of an individual with just one measurement, their timing random effect is zero). Thus the central groups have fewer measurements per subject on average, and also less opportunity to generate residual variation.

It is noticeable with the predicted velocity curves (Figures 9 and 10, bottom left) that PV falls steadily as APV rises; this is due to the SITAR model using log age rather than age, which provides a better fit as APV is negatively correlated with PV as previously discussed (Cole et al. 2014). Tanner also documented a negative correlation between APV and PV in his Harpenden analysis (Tanner et al. 1976). This same trend can be seen with the locally estimated velocity curves (bottom right), particularly in boys, though the curves are more ragged. Note that in girls the agreement between the global and local velocity curves is generally close.

However for boys two features of the local velocity curves are absent from the global curves – the later APV groups show a peak in velocity at around 8.5 years that the earlier APV groups lack, and several of the curves dip below zero velocity approaching age 20, indicating predicted mean adult height falling over time. The early velocity peak is also seen in the height curves (top right, Figure 10) as an upward bulge at age 9, restricted to the older APV groups. The two features occur at the extremes of age and show that SITAR is less effective at modelling these extremes.

The negative velocity curves may arise from the timing of the final data sweep at age 17. It is clear from Figure 7 that the data are sparse after age 18.5, and it would require only slight downward bias to tip the mean curve slopes negative.

**Figure 12.**
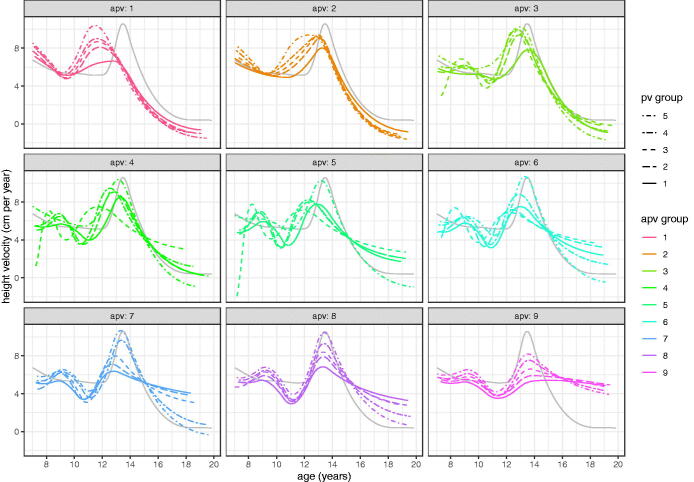
SITAR mean height velocity curves for ALSPAC boys, split into nine groups by age at peak height velocity (APV) and in turn five groups by peak height velocity (PV).

Figures 11 (girls) and Figures 12 (boys) show each APV group split into five PV groups. The individual girls curves are generally similar in shape, although a few dip below zero velocity at age 19. However the boys curves show a remarkable spectrum of curve shapes, ranging from the earliest and fastest growing (top curve, top left facet) to the latest and slowest growing (bottom curve, bottom right facet).

The peak in velocity at age 9, seen consistently in the later APV groups of Figure 12, looks like a midgrowth spurt, but this term has already been applied to a spurt documented as occurring nearer age 7 than 9 (Tanner and Cameron 1980; Gasser et al. 1985; Remer and Manz 2001). The data analysed here start at age 7 and so are not able to detect a spurt that early. Whether this age 9 peak is just a delayed version of the age 7 peak, or whether it is novel, would need further analysis to unpick.

**Figure 13.**
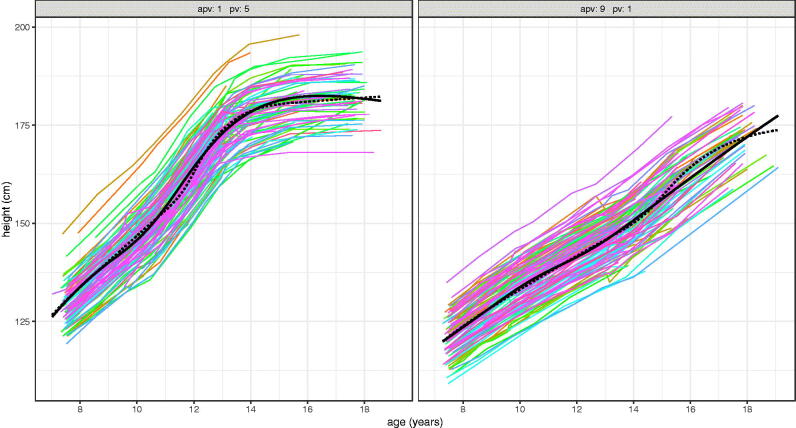
Height growth curves for ALSPAC boys with two contrasting patterns of development; left) early and fast - APV group 1 and PV group 5 (*n* = 116), and right) late and slow – APV group 9 and PV group 1 (*n* = 117). The corresponding SITAR mean curves are shown as solid black lines, and the dotted lines are predicted mean curves (based on mean APV and mean PV) from the global SITAR model.

To demonstrate the spectrum of height growth in boys, Figure 13 shows the individual growth curves in the two extreme timing-intensity groups of Figure 12, each representing 2.2% of the cohort total. Their mean curves are also shown superimposed (with solid lines for the local mean and dotted lines for the predicted global mean). Because they are filtered by APV and PV the individual curves are essentially parallel to each other. Mean APV in the two groups is 11.5 and 15.0 years respectively, i.e. 3.5 years apart, while mean PV is 10.4 and 5.4 cm per year, a factor of two different (Figure 12). Yet despite these differences SITAR does a reasonable job of predicting the two growth patterns from the global model (dotted lines), although it infers a growth spurt in the late curve where none exists.

This ability of SITAR to identify groups of individuals with early or late APV could be exploited in cohort studies to construct specialist growth references for early and late developers. It could be done for example using all the data for the first and last groups, respectively, of Figures 9 and 10 (*n* > 4000, Table 5), and fitting growth reference centiles to them using the LMS method (Cole and Green 1992). This would be most useful for the clinical management of constitutional growth delay, where the chart would document the growth pattern of the 11% most delayed normal children. It might also be reassuring to parents to see just how late the growth spurt can be.

However Figure 13 makes clear that the late developing boys are still growing at age 19, so there would be insufficient data to extend the centiles to final height. And it should be acknowledged that Tanner staging could be used instead of APV to identify the early and late groups. However APV is likely to be more accurate, being based on a growth curve rather than a single clinical assessment, and measured on a continuous rather than a five-point scale.

### Strengths and limitations

The study has some obvious strengths. The Harpenden Growth Study is an important resource of very high quality growth data containing information on ten separate measurements in a cohort of children born seventy years ago, while ALSPAC provides high quality height data on a very large number of individuals born thirty years ago. Given this richness of material it is hard to identify limitations in the study design, and the analysis was thorough, though it could obviously have been extended in a number of ways.

## Conclusions

The analysis of growth data during puberty is greatly simplified using SITAR, as the subject random effects of timing and intensity accurately characterise the tempo of growth of individuals. The analyses of data from the Harpenden Growth Study and ALSPAC have shed light on the growth patterns of a range of linear measurements and on the spectrum of height growth as seen in early, average and late developers.

## Supplementary Material

Supplemental Material

## Data Availability

The Harpenden Growth Study data were kindly made available by Professor Noël Cameron (Loughborough University). The ALSPAC data were made available courtesy of the ALSPAC Executive Committee (project proposal B1036).
