# Supplementary material for: Tanner’s tempo of growth in adolescence: recent SITAR insights with the Harpenden Growth Study and ALSPAC
Source: Ann Hum Biol. 2020 May 20;47(2):181–98. doi: 10.1080/03014460.2020.1717615 (PMC7391859; doi:10.1080/03014460.2020.1717615)
Supplement: Supplemental Material [file IAHB_A_1717615_SM0750.pdf]

Supplementary Table 1. Harpenden Growth Study correlations of individual random effects across measurements, by sex.

**Boys size**

| | height | leg | foot | sitting | shoulder | hip | knee | elbow | thigh | arm |
|---|---|---|---|---|---|---|---|---|---|---|
| height | | | | | | | | | | |
| leg | 0.87 | | | | | | | | | |
| foot | 0.72 | 0.67 | | | | | | | | |
| sitting | 0.77 | 0.50 | 0.61 | | | | | | | |
| shoulder | 0.65 | 0.52 | 0.49 | 0.56 | | | | | | |
| hip | 0.47 | 0.39 | 0.41 | 0.50 | 0.38 | | | | | |
| knee | 0.52 | 0.43 | 0.53 | 0.55 | 0.48 | 0.51 | | | | |
| elbow | 0.50 | 0.40 | 0.51 | 0.49 | 0.55 | 0.40 | 0.62 | | | |
| thigh | 0.30 | 0.21 | 0.29 | 0.38 | 0.45 | 0.39 | 0.52 | 0.60 | | |
| arm | 0.16 | 0.07 | 0.24 | 0.28 | 0.37 | 0.20 | 0.42 | 0.50 | 0.72 | |

**Boys timing**

| | height | leg | foot | hip | sitting | shoulder | elbow | knee | arm | thigh |
|---|---|---|---|---|---|---|---|---|---|---|
| height | | | | | | | | | | |
| leg | 0.76 | | | | | | | | | |
| foot | 0.63 | 0.63 | | | | | | | | |
| hip | 0.67 | 0.69 | 0.56 | | | | | | | |
| sitting | 0.71 | 0.53 | 0.54 | 0.75 | | | | | | |
| shoulder | 0.69 | 0.54 | 0.59 | 0.55 | 0.53 | | | | | |
| elbow | 0.62 | 0.56 | 0.56 | 0.52 | 0.51 | 0.51 | | | | |
| knee | 0.45 | 0.41 | 0.45 | 0.43 | 0.38 | 0.37 | 0.46 | | | |
| arm | 0.27 | 0.19 | 0.31 | 0.31 | 0.35 | 0.24 | 0.22 | 0.24 | | |
| thigh | 0.09 | 0.10 | 0.07 | 0.21 | 0.15 | 0.16 | 0.15 | 0.16 | 0.47 | |

**Boys intensity**

| | height | leg | sitting | foot | elbow | knee | hip | shoulder | thigh | arm |
|---|---|---|---|---|---|---|---|---|---|---|
| height | | | | | | | | | | |
| leg | 0.73 | | | | | | | | | |
| sitting | 0.68 | 0.29 | | | | | | | | |
| foot | 0.44 | 0.42 | 0.32 | | | | | | | |
| elbow | 0.46 | 0.32 | 0.43 | 0.46 | | | | | | |
| knee | 0.51 | 0.41 | 0.42 | 0.49 | 0.62 | | | | | |
| hip | 0.42 | 0.37 | 0.39 | 0.29 | 0.34 | 0.38 | | | | |
| shoulder | 0.49 | 0.37 | 0.38 | 0.34 | 0.40 | 0.42 | 0.33 | | | |
| thigh | 0.14 | 0.10 | 0.16 | 0.09 | 0.30 | 0.44 | 0.39 | 0.34 | | |
| arm | 0.06 | -0.04 | 0.11 | 0.10 | 0.22 | 0.27 | 0.18 | 0.35 | 0.71 | |

**Girls size**

| | height | leg | foot | sitting | shoulder | elbow | knee | hip | thigh | arm |
|---|---|---|---|---|---|---|---|---|---|---|
| height | | | | | | | | | | |
| leg | 0.87 | | | | | | | | | |
| foot | 0.72 | 0.71 | | | | | | | | |
| sitting | 0.80 | 0.54 | 0.58 | | | | | | | |
| shoulder | 0.66 | 0.59 | 0.60 | 0.59 | | | | | | |
| elbow | 0.52 | 0.45 | 0.52 | 0.53 | 0.51 | | | | | |
| knee | 0.46 | 0.34 | 0.49 | 0.54 | 0.45 | 0.59 | | | | |
| hip | 0.34 | 0.22 | 0.24 | 0.45 | 0.40 | 0.33 | 0.45 | | | |
| thigh | 0.25 | 0.18 | 0.26 | 0.33 | 0.35 | 0.43 | 0.51 | 0.38 | | |
| arm | 0.08 | 0.08 | 0.16 | 0.12 | 0.16 | 0.40 | 0.40 | 0.25 | 0.71 | |

**Girls timing**

| | height | leg | sitting | foot | shoulder | hip | elbow | knee | thigh | arm |
|---|---|---|---|---|---|---|---|---|---|---|
| height | | | | | | | | | | |
| leg | 0.83 | | | | | | | | | |
| sitting | 0.80 | 0.66 | | | | | | | | |
| foot | 0.68 | 0.74 | 0.71 | | | | | | | |
| shoulder | 0.73 | 0.68 | 0.76 | 0.66 | | | | | | |
| hip | 0.65 | 0.63 | 0.69 | 0.62 | 0.65 | | | | | |
| elbow | 0.48 | 0.48 | 0.56 | 0.51 | 0.59 | 0.57 | | | | |
| knee | 0.34 | 0.37 | 0.40 | 0.49 | 0.35 | 0.50 | 0.5 | | | |
| thigh | 0.16 | 0.23 | 0.31 | 0.28 | 0.46 | 0.35 | 0.51 | 0.38 | | |
| arm | 0.02 | 0.15 | 0.16 | 0.29 | 0.26 | 0.22 | 0.42 | 0.43 | 0.70 | |

**Girls intensity**

| | height | leg | knee | sitting | elbow | foot | hip | shoulder | thigh | arm |
|---|---|---|---|---|---|---|---|---|---|---|
| height | | | | | | | | | | |
| leg | 0.75 | | | | | | | | | |
| knee | 0.32 | 0.23 | | | | | | | | |
| sitting | 0.67 | 0.19 | 0.18 | | | | | | | |
| elbow | 0.24 | 0.14 | 0.50 | 0.20 | | | | | | |
| foot | 0.32 | 0.32 | 0.31 | 0.15 | 0.23 | | | | | |
| hip | 0.40 | 0.24 | 0.47 | 0.45 | 0.36 | 0.24 | | | | |
| shoulder | 0.35 | 0.23 | 0.12 | 0.36 | 0.12 | 0.17 | 0.37 | | | |
| thigh | 0.11 | 0.05 | 0.15 | 0.19 | 0.04 | 0.14 | 0.29 | 0.28 | | |
| arm | 0.01 | 0.04 | 0.03 | 0.10 | 0.04 | 0.11 | 0.20 | 0.15 | 0.65 | |
